# Unique Use of Dibromo–L–Tyrosine Ligand in Building of Cu(II) Coordination Polymer—Experimental and Theoretical Investigations

**DOI:** 10.3390/molecules29112709

**Published:** 2024-06-06

**Authors:** Agnieszka Wojciechowska, Jan Janczak, Tomasz Rojek, Muhammad Ashfaq, Magdalena Malik, Natasza Trzęsowska, Rafał Wysokiński, Julia Jezierska

**Affiliations:** 1 Faculty of Chemistry, Wrocław University of Science and Technology, Wyb. Wyspiańskiego 27, 50-370 Wrocław, Poland; tomasz.rojek@pwr.edu.pl (T.R.); magdalena.malik@pwr.edu.pl (M.M.); natasza.trzesowska@pwr.edu.pl (N.T.); rafal.wysokinski@pwr.edu.pl (R.W.); 2 Institute of Low Temperature and Structure Research, Polish Academy of Sciences, Okólna 2, 50-422 Wrocław, Poland; j.janczak@intibs.pl; 3 Department of Physics, University of Sargodha, Sargodha 40100, Pakistan; ashfaq.muhammad@uos.edu.pk; 4 Faculty of Chemistry, University of Wrocław, Joliot-Curie 14, 50-383 Wrocław, Poland; julia.jezierska@uwr.edu.pl

**Keywords:** 3,5–dibromo–L–tyrosine, copper(II), crystal structure, spectroscopies, theoretical calculations

## Abstract

Although the crystals of coordination polymer {[CuCl(*μ-*O,O’-L-Br_2_Tyr)]}n (1) (L-Br_2_Tyr = 3,5-dibromo-L-tyrosine) were formed under basic conditions, crystallographic studies revealed that the OH group of the ligand remained protonated. Two adjacent [CuCl(L-Br_2_Tyr)] monomers, bridged by the carboxylate group of the ligand in the *syn-anti* bidentate bridging mode, are differently oriented to form a polymeric chain; this specific bridging was detected also by FT-IR and EPR spectroscopy. Each Cu(II) ion in polymeric compound 1 is coordinated in the xy plane by the amino nitrogen and carboxyl oxygen of the parent ligand and the oxygen of the carboxyl group from the symmetry related ligand of the adjacent [Cu(L-Br_2_Tyr)Cl] monomer, as well as an independent chlorine ion. In addition, the Cu(II) ion in the polymer chain participates in long-distance intermolecular contacts with the oxygen and bromine atoms of the ligands located in the adjacent chains; these intramolecular contacts were also supported by NCI and NBO quantum chemical calculations and Hirshfeld surface analysis. The resulting elongated octahedral geometry based on the [CuCl(L-Br_2_Tyr)] monomer has a lower than axial symmetry, which is also reflected in the symmetry of the calculated molecular EPR g tensor. Consequently, the components of the d-d band obtained by analysis of the NIR-VIS-UV spectrum were assigned to the corresponding electronic transitions.

## 1. Introduction

L–Tyrosine (L–Tyr; 2–amino–3–(4–hydroxyphenyl)propanoic acid) and its derivatives show very good coordination properties and can bind to metal ions, forming complexes in solution or in the solid state [1,2,3,4,5,6]. L–Tyr can form the numerous derivatives mostly based on the mono- or di- substitution of the phenolic ring in positions 3- or 3, 5-, respectively, e.g., by nitro, methoxy, amino, and halogen substituents [7]. One of the most common modes of L–Tyr and its derivatives coordination is a bidentate chelation of metal ions via amine nitrogen and one carboxylate oxygen, resulting in typically coordinated metal monomers. As is well known, the amino acid carboxyl group, due to its bidentate nature, can bridge two adjacent metal ions by two carboxylate oxygens leading to dimeric or polymeric compounds [5,8,9]. In agreement with the crystal structure of the Cu(II) complex with L–Tyr ligands, one of the oxygen atoms of the *μ*_2_-carboxylate group of the ligand, not involved in ON-type Cu(II) chelation, connects neighboring Cu(II) ions, forming a right-handed chiral 1D chain structure of [Cu(L–Tyr)_2_]_n_ [10,11,12]. The l–TyrZn, l–TyrCo/Zn, and l–TyrCo systems are isomorphous chiral neutral 3D coordination polymers (MOF). As usual, each of the two *μ-*carboxylates can bridge two metal ions, and four independent metal ions are bridged and coordinated by two L-Tyr ligands [13]. Another interesting derivative of L-Tyr is poly[diaqua-*μ*–4,4′–bipyridine–di–nitrato–di–*μ–*l–tyrosinato–dicopper(II)], where adjacent Cu atoms are bridged by bidentate carboxylate groups into a chain [14].

The 3–I or 3, 5–I_2_ l–Tyr derivatives show very low solubility; therefore, crystal structure and other physicochemical studies of their metal ion complexes are limited. The crystal structures revealed that two Cu(II) ions in complexes with l–I_2_Tyr are connected by the carboxylate group of the ligand to form 1D chains of {[CuCl(*μ–O,O’*–l–I_2_Tyr)]}_n_ [15]. Interestingly, it was found in 1D chain of {[CuCl(*μ–O,O’*–l–I_2_Tyr)]}_n_ that the axial positions of Cu(II) are involved in intermolecular weak interactions with iodine (Cu···I = 3.248(4) Å) and phenolic oxygen atoms (Cu···O = 3.22(4) Å) from the l–I_2_Tyr ligands located in adjacent chains [15]. Previously, these Cu···I and Cu···O_phenolic_ interactions were observed in the change in proton NMR signals upon adding Cu(II) ions to the solution of the 3, 5–dimethyltyrosine (I_2_Tyr) and 4–methoxy–3,5–di–methyltyrosine ((CH_3_)_2_TyrOCH_3_) [16].

The crystalographically characterized mixed ligand copper(II) complexes with diiodo–l–tyrosinate, i.e., [Cu(l–I_2_Tyr)(bpy)(NO_3_)]∙CH_3_OH [17], [Cu(l–I_2_tyrO^−^)(bpy)(H_2_O)]∙2H_2_O [17], [Cu(l–ITyr)(bpy)(H_2_O)]∙NO_3_∙CH_3_OH∙H_2_O [18], [Cu(l–I_2_TyrO^−^)(phen)(H_2_O)]∙2H_2_O [19], [Cu(l–I_2_Tyr)(phen)(H_2_O)]∙NO_3_ [20], and [Cu(l–I_2_Tyr)(phen)Cl]∙2H_2_O [20], [Cu(l–I_2_tyrO^−^)(hista)(H_2_O)]_2_∙2H_2_O [18], are by far monomers with relatively long Cu···Cu distances of over ca. 6 Å. Thus, three of these complexes, synthesized in the basic solution, contain a phenolate group in the l–I_2_tyrO^−^ ligand [17,18,19].

How difficult and rare it was to obtain a crystalline metal complex with 3, 5–dibromosubstituted L–tyrosine is evidenced by the fact that only one of that type of crystal structure, i.e., [Ni(phen)(l–Br_2_Tyr)_2_·2CH_3_OH·2H_2_O] [21], is deposited in Cambridge Structural Database (CSD) [22].

In this work, we present the successful preparation of crystals suitable for the X-ray measurement and full structure determination of the first example of coordination polymer based on 3,5–dibromo–l–tyrosine with a carboxylate group acting as a bidentate bridge between two Cu(II) ions. The in-depth crystal structure analysis was combined with spectroscopic (FT–IR, FT–Raman, X- and Q-band EPR, and NIR–Vis–UV) methods. Quantum chemistry QTAIM, NCI, and NBO computational methods were used to investigate in detail the nature of interactions in the coordination sphere. In addition, Hirshfeld surface analysis in correlation with X-ray data allowed the packing of crystal 1 to be estimated in terms of the share of short and long interatomic contacts, hydrogen bonds and stacking between phenolic rings.

## 2. Results and Discussion

### 2.1. Crystal Structure Description of 1

**Figure 1:**
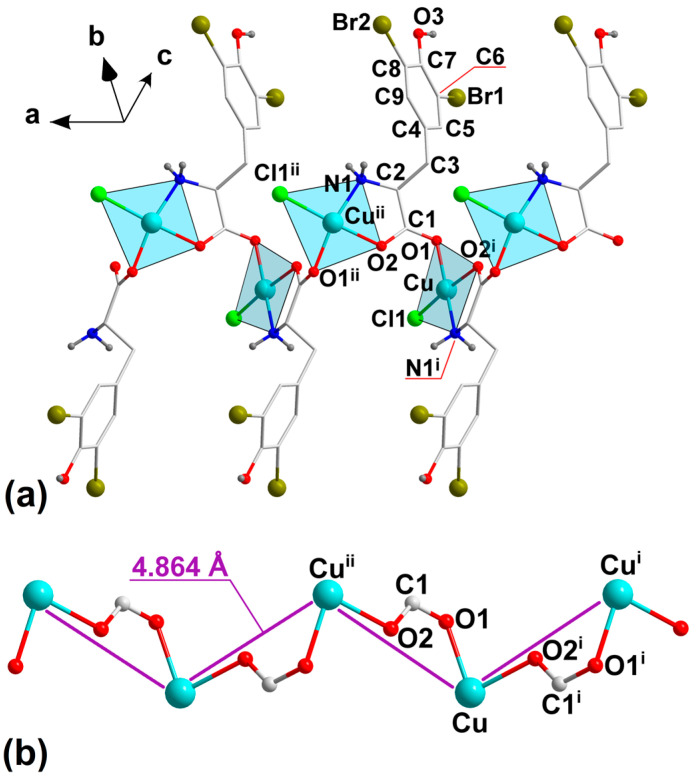
(**a**) 1D polymeric chain of **1** with atom numbering scheme. (**b**) Connectivity of Cu(II) centers within 1D chain by means of O–C–O bridge. All C–bounded H–atoms are omitted for clarity in picture (**a**). Symmetry codes: (i) *x* − 1/2, −*y* + 1/2, −*z* + 1, (ii) *x* + 1/2, −*y* + 1/2, −*z* + 1.

A dibromo-functionalized l–tyrosine with Cu(II) ions are connected to form a 1D coordination polymer represented by the formula {[Cu(l–Br_2_Tyr)Cl]}_n_ (**1**). Table S1 presents data collection parameters, crystallographic data, and final agreement parameters. The selected intermolecular contacts with bond angles, and the proposed hydrogen bonds, contacts, and intramolecular interactions are shown in Table 1 and Table 2, respectively. The asymmetric unit of complex **1** consists of one Cu(II) ion, one l–Br_2_Tyr ligand with a protonated hydroxyl group, and one coordinated chloride ion (Figure 1a and Figure S1).

The presence of a proton on the hydroxyl group is indicated by the length of the C–OH bond, which is 1.360(6) Å in **1**. The C–OH and deprotonated C–O^−^ groups were structurally characterized by single-crystal X-ray diffractions for brominated bisphenol derivatives (containing dibromo-4-hydroxyphenyl rings) with organoamines. It was found that the C-O^−^ bond is much shorter (average value of 1.300 Å), while the C–OH bond (average value of 1.360 Å) [23] is similar to that for **1**.

An extensive search of the Cambridge Structural Database (CSD v. 5.4.1 [22]; ConQuest v. 2020.1 [24]) for Cu(II) metal complexes based on l–Tyr and its derivatives revealed a {[Cu(l–I_2_Tyr)Cl]}_n_ complex reported by *Okabe* and *Hokaze*, which displays a polymeric architecture similar to **1** [15].

The smaller atomic radius of bromine than iodine placed in the aromatic group of l–Tyr is reflected in the shorter C–Br bond length (C6–Br1 = 1.907(5) Å and C8–Br2 = 1.896(5) Å) in **1** compared to C–I (C3–I1 = 2.082 Å and C5–I2 = 2.189 Å) in {[Cu(l–I_2_Tyr)Cl]}_n_ (Table 1) [15]. Similarly to {[Cu(l–I_2_Tyr)Cl]}_n_, the Cu(II) cation in compound **1** is coordinated in the *xy* plane by nitrogen amino N1^i^ and carboxyl O2^i^ atoms from parent ligand and carboxyl O1 atom from the symmetry related ligand of the adjacent [Cu(l–I_2_Tyr)Cl] monomer as well as by an independent Cl1 chlorine ion (Figure 1a).

The observed in-plane Cu–O and Cu–N bond distances are within the expected and narrow range of 1.9–2.0 Å and correspond to those found in {[Cu(l–I_2_Tyr)Cl]}_n_ (Cu–O = 1.97(2) Å and 2.00(2) Å, Cu–N = 1.97(3) Å), as well as in other Cu(II) complexes based on l–Tyr amino acid or its iodo-derivatives, such as [Cu(l–TyrOH)(nphen)(H_2_O)]NO_3_·H_2_O (nphen = 5–nitro–1,10–phenanthroline) [25], [Cu(l–TyrOH)(4–mphen)(H_2_O)]ClO_4_ (4–mphen = 4–methyl–1,10–phenantroline) [26], and [Cu(l–ITyrOH)(bpy)(H_2_O)]NO_3_·CH_3_OH·H_2_O [18] or [Cu(l–I_2_TyrO^−^)(bpy)(H_2_O)]·H_2_O [17]. The Cl^−^ anion that is the most distant from the metal center coordinates with the Cu–Cl1 bond length of 2.2217(15) Å and 2.231(8) Å in **1** and {[Cu(l–I_2_Tyr)Cl]}_n_ [15], respectively. As expected, the Cu–Cl bond is longer than those of the Cu–O and Cu–N due to the larger ionic radius of the Cl^−^ anion. These distances for the in-plane Cu–Cl bond correspond to those observed in [Cu_4_(l–Arg)_4_Cl_8_] (in the range of 2.238–2.287 Å) [27], [Cu(l–Hist)Cl_2_] (2.254 Å and 2.293 Å) [28], and [Cu(l–Hist)Cl_2_]·0.5H_2_O (2.246 Å and 2.300 Å) [29]. Apart from the indicated differences in the bond lengths, the analysis is complemented by the comparison of in-plane bond angles. Herein, the effect of the larger ionic radius of the Cl^−^ anion than O and N atoms results in larger N1^i^–Cu–Cl1 and O1–Cu–Cl1 bond angles of ca. 95–97° than N1^i^–Cu–O2^i^ and O1–Cu–O2^i^ of ca. 83–86°. The trans- N1^i^–Cu–O1 and O2–Cu–Cl1 bond angles are 164.5° and 175.1°, respectively. Consequently, the Cu(II) ion deviates slightly by 0.045 Å from the average plane defined by coordinated atoms.

The carboxyl O1 atom is involved in the monodentate binding of the Cu(II) ion while the carboxyl O2 and amino N1 atoms chelate one symmetry-related Cu^ii^ ion. As a result, the pairs of Cu and Cu^ii^ cations are connected through a single *syn-anti* O1–C1–O2 carboxylate bridge with a Cu···Cu^ii^ distance of 4.864 Å (Figure 1a,b).

The O1–C1–O2 carboxyl bridges link the coordination units related by the two-fold screw axis parallel to the [100] direction to form one dimensional (1D) coordinating polymer running along the crystallographic *a*–axis. A similar arrangement of the building units into infinite 1D structures is also observed in {[Cu(l–I_2_Tyr)Cl]}_n_ with a Cu···Cu distance of 4.949 Å within the chain. Besides the isomorphous {[Cu(l–I_2_Tyr)Cl]}_n_ complex, only the [Cu(l–Tyr)_2_]_n_ complex is recognized as higher dimensional coordination polymers, and the structure is organized into a 1D chiral chain through exobidentate O–C–O carboxyl bridge [10,11,12].

**Figure 2:**
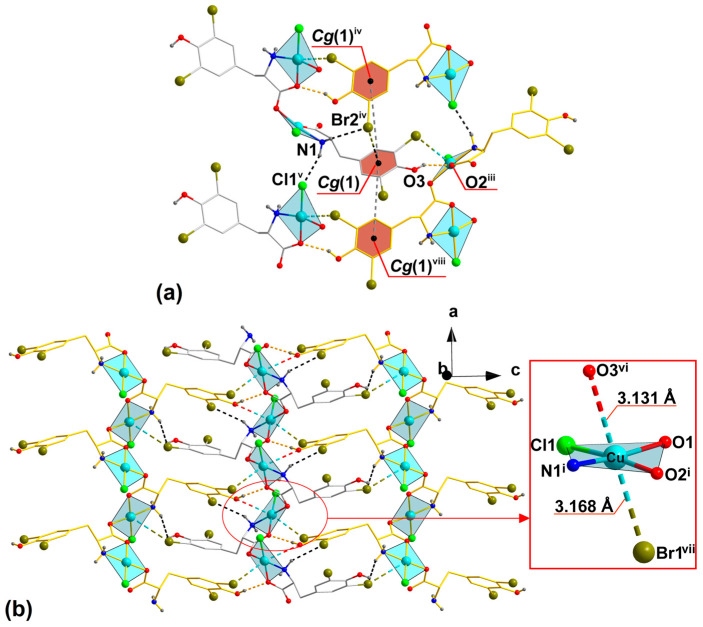
Crystal packing of **1**. (**a**) The role of O–H···O hydrogen bonds (light orange dashed lines) and weak N–H···Cl, N–H···Br, as well as *Cg*···Br and *Cg*···*Cg* contacts (black dashed lines) in connection with adjacent 1D coordination polymers in a 3D supramolecular network. (**b**) The packing of the 1D polymer in the unit cell showing hydrogen bonds and the electrostatic interaction (dashed line) between the square coordinated Cu center of polymer with the Br1 and O3 atoms of neighboring 1D polymers. The position of O3^vi^ and Br1^vii^ atoms relative to Cu(II) center. All C-bounded H-atoms are omitted for clarity. Symmetry codes are given in Table 2.

In **1**, the coordination chains are further extended into a 3D supramolecular network by means of O3–H3···O2^iii^ (*D*···*A* = 2.804(5) Å) hydrogen bonds established between the hydroxyl O3 atom and the carboxyl O2^iii^ atom from the symmetry-related ligand (Figure 2a, Table 2). The stabilization of the network is realized with different types of weak interactions. The N1–H1*A*···Br2^iv^ (*D*···*A* = 3.651(4) Å) and N1–H1*B*···Cl1^v^ (*D*···*A* = 3.280(5) Å) contacts are generated between the amino group and the coordinated Cl1 ion, together with the Br2 atom belonging to the l–Br_2_Tyr ligand, respectively. The [C4–C5–C6–C7–C8–C9] *Cg*(1) aromatic ring of l–Br_2_Tyr moiety forms contact with the Br2^iv^ atom *Cg*(1)···Br2^iv^, with a distance of 3.450(2) Å. These contacts are generated with the aromatic rings from adjacent 1D chain interactions *Cg*(1)···*Cg*(1)^iv^ and *Cg*(1)···*Cg*(1)^viii^ of 5.798(3) and 5.161(3) Å, respectively (Figure 2a and Figure S2). Additionally, between adjacent 1D coordination polymers, there are the intermolecular long-distance O3^vi^ ··Cu(II)··Br1^vii^ contacts {Cu···Br1^vii^ = 3.1677 (8) Å and Cu···O3^vi^ = 3.131(3) Å] between the Cu(II) centers and bromine and oxygen substituents of L-Br_2_Tyr ligands from these adjacent polymers (Figure 2b).

In the case of Cu(II) complexes, noncovalent intermolecular contacts and semi-coordination interactions at distances greater than 3 Å are known. Thus, the Cu···O contact in the axial position of copper has been found at a distance close to 3.2 Å [15], similar to our complex **1**, and even at a much larger distance (about 3.6 Å), this contact mediated magnetic super-exchange interactions in another Cu(II) complex [30]. The Cu(II) ion in the CuN_4_O_4_ complex was partially coordinated by four carbonyl oxygen atoms at distances of about 2.8 to 3.0 Å [31]. Cu···Br contacts (at a Cu–Br distance close to 3.2 Å) have been observed in axial positions in the CuN_2_Br_4_ octahedron [32] and at distances close to 3.1 Å in the Cu(II)–tetrazole complex, where they act as bridges involved in ferromagnetic interactions [33]. Complex **1** can, therefore, be considered as an octahedron, but with symmetry deviated from the axial one. This is evidenced by *cis-* and *trans*-bonds formed between Cu(II) ions, apical Br1^vii^ and O3^vi^ atoms, and in-plane NOO’Cl donors. The *cis* bond angle, which remains in the range of 66.7–103.8° and 76.3–105.0°, respectively, and the *trans* bond angle of 167.9°, are significantly different from 90° (for *cis*) and 180° (for *trans*) bonds.

The system of O–H···O, N–H···Cl and N–H···I hydrogen bonds found in the 3D supramolecular architecture of the previously reported {[Cu(l–I_2_Tyr)Cl]}_n_ compound is analogous to that of the isomorphous complex **1** only if the N–H···I is replaced by N–H···Br Another important finding is that the hydroxyl oxygen and the iodine atoms from the l–I_2_Tyr ligands are in a similar orientation, position, and distances relative to Cu(II) (3.218 Å and 3.248 Å, respectively) [15]. Therefore, both iodine and oxygen atoms are involved in the stabilization crystal packing by *Cg*···I, Cu···O, and Cu···I analogously to *Cg*···Br, Cu···O, and Cu···Br contacts revealed by us for compound **1**.

### 2.2. Theoretical Quantum Chemistry AIM, NCI, and NBO Calculations

**Figure 3:**
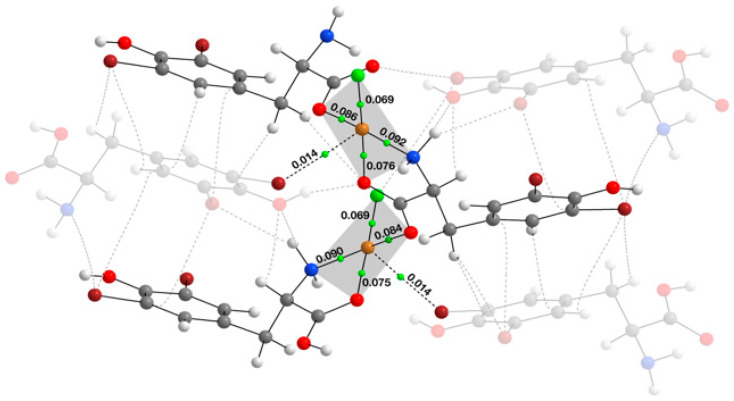
The results of AIM calculations performed for the model fragment of {[CuCl(*μ–O,O’*–l–Br_2_Tyr)]}_n_ complex shown in Scheme S1.

Theoretical calculations were performed on the fragment of the **1** polymer structure cut out from the crystal lattice (Scheme S1). QTAIM analysis operates through the electron density topology of a molecule; it determines the bond critical points (BCPs) corresponding to the saddle points of the electron density gradient. These saddle points define the chemical bonds between atoms and represent the minimum electron density along the bond direction and its maxima in all others [34]. The QTAIM technique was employed to characterize the first coordination sphere around Cu(II) in compound **1**. Figure 3 illustrates the outcomes of the analysis for the model under consideration. The primary polymer chain and the investigated interactions are highlighted for clarity.

Bond critical points (BCPs) are depicted as green dots in the middle of the bond paths. The electron density value (ρ) between copper and ligands in BCPs ranges from 0.069 (Cu–Cl) up to 0.092 a.u. (Cu–N) (Table 3). The AIM analysis also revealed the noncovalent interaction between copper and bromine atoms (Cu∙∙∙Br) with a ρ value of 0.014 a.u. Espinosa’s equation [35] for weak interactions enables an estimation of the interaction energy (E_int_) for Cu∙∙∙Br, with the value of E_int_= −6.28 kcal/mol. In all cases, the Laplacian of the electron density (∇^2^ρ) takes values greater than zero, which indicates electron density depletion, and qualifies these bonds into “closed-shell” interactions [36]. The total energy density (H) at the BCPs, being the sum of the kinetic (G) and potential (V) energies, takes negative values. In conclusion, low values of electron density (ρ < 0.1), the values of G and |V| energies together with positive values of ∇^2^ρ, and total energy density (H) values close to zero are indicative of donor–acceptor bond characteristics [36].

**Figure 4:**
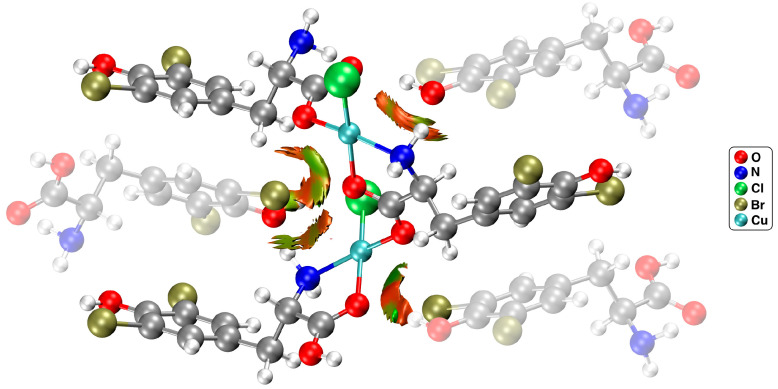
NCI isosurfaces for the model fragment of {[CuCl(*μ–O*,*O*’–l–Br_2_Tyr)]}_n_ complex at the RDG 0.5 a.u. isovalue (green surfaces represent noncovalent interaction regions).

The complementary non-covalent index (NCI) analysis allowed for a qualitative evaluation of the QAIM results. The NCI provides a visual representation of the interactions within the studied structure, presenting them as isosurface positioned between interacting atoms (Figure 4: the main chain is highlighted to enhance transparency).

The green areas represent noncovalent interactions and are visible between the Cu and Br atoms, as well as between the copper center and the phenolic OH group. The outcome exhibits that the second interaction is significantly weaker, as evidenced by its less intense green hue and smaller isosurface size.

According to the NBO analysis, in-plane interactions with nitrogen and oxygen atoms are designated as coordinate bonds; the Cu–Cl bond, however, exhibits a covalent character doped by donor–acceptor interactions. Among coordinate bonds, those with oxygens are the strongest with similar E^2^ values of around 70 kcal/mol (Table 4).

The interaction between nitrogen and copper is slightly weaker (62.89 kcal/mol). The NBO highlighted two more interactions in the axial position with the bromine and oxygen atoms of the phenolic hydroxyl group (Figure S4).

The second-order energy values reveal a 7-fold decrease in the case of interaction between copper and bromine. The interaction with the oxygen atom of the hydroxyl group, the weakest among those described earlier, completes the octahedral environment of the copper atom. Figure S4 depicts the overlap between a lone pair on the bromine and oxygen atoms and a lone vacant (LV) orbital on the Cu of the other chain. Additional information on the coordination sphere can be found in Table S2.

The results from the computational methods (AIM, NCI, and NBO) described above made it possible to investigate the first coordination sphere with an emphasis on weak interactions. NCI and NBO clearly showed interactions with both the bromine and hydroxyl groups. AIM analysis suggested only Cu∙∙∙Br interaction; however, there are cases in the literature where the AIM methodology failed to identify bonding paths [37].

### 2.3. Hirshfeld Surface Analysis

**Figure 5:**
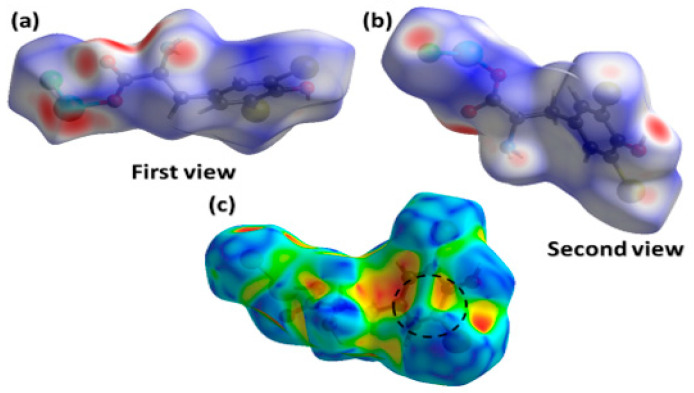
Hirshfeld surface plotted over (**a**,**b**) normalized distances; (**c**) shape index for **1**.

We have used Hirshfeld surface analysis (HSA) to calculate and visualize intermolecular (non-covalent) atomic contacts in the complex **1** crystal, demonstrated by normalized contact distances (d*_norm_*) mapped onto 3D Hirshfeld surfaces (HS). The d*_norm_* is based on the sum of two distances between the nearest atoms, inside (d*_i_*) and outside (d*_e_*), with respect to the van der Waals (vdW) radii of the same atoms. Intermolecular forces are color-coded on HS, where distances that are shorter than the sum of the vdW radii are shown in red (Figure 5a,b) while the longer ones are shown in blue [38]. In the case of **1**, the crystal packing is arranged by a single molecule that extends to infinity in a 3D supramolecular network. The asymmetric unit was used.

The distances d_e_ and d_i_ plotted on the Hirshfeld surface give a 3D presentation of intermolecular contacts in a crystal. The summary fingerprint plot is next decomposed into the fingerprint plots for particular pairs of atoms involved in the intermolecular contacts and their percentage to the total HS are calculated [39]. The fingerprint plots generated for compound **1** are shown in Figure S5 and Figure S6 and the contact percentages are collected in Table S3. 

Below each fingerprint plot, the share of the same contacts is shown by blue regions on the corresponding surface. The relative prosperity of the particular contact in the crystal is reflected by its enrichment ratio E_xy_, which is a ratio between the percentage of the actual X∙∙∙Y contacts and the percentage of random contacts, calculated under conditions that all types of X∙∙∙Y of contacts had the same probability of forming in the crystal (Table S3) [40]. Enrichment ratios greater than 1 for X∙∙∙Y contacts indicate that these contacts are preferred in the crystal. The calculations based on HSA in correlation with the crystal structure data (Table 2) revealed the following characteristics of major intermolecular contacts in the crystal packing of **1:** (a) red colored spots on HS close to the copper atom represent Cu∙∙∙O and Cu∙∙∙Br contacts, with a contribution of 4% and 2.3%; E_CuO_ = 2.55 (the highest) and E_CuBr_ = 1.0; these intermolecular contacts occur in the crystal structure. (b) Red color spots on HSs close to the NH_2_ group whose hydrogens participate in H∙∙∙Cl and H∙∙∙Br (lighter) contacts have contributions of 17% and 22.9% (the most abundant), with E_HCl_ = 1.95 and E_HBr_ = 1.52 respectively; these contacts are associated with the N1–H1···Cl1 (shorter) and N1–H1···Br2 (longer) hydrogen bonds. (c) Dark red spots on HSs close to carboxylate oxygen and phenolic oxygen have contributions of 17%, and E_HO_ = 1.7; the contacts are connected with O3–H∙∙∙O2 hydrogen bonds in the crystal structure. d) Blue regions in the fingerprint plot represent C∙∙∙Br contacts, with a contribution of 7.4% and E_CBr_ = 1.8; this was also found in the crystal packing. The red (concave) and blue (convex) areas visible in the dashed circle on the HS mapped with the shape index (Figure 5c) can be attributed to the stacking interactions between the phenolic rings. According to the crystal structure, the rings are located at large distances of 5.798 and 5.165 Å for identical monomeric units of **1** (Figure S2) and at an angle of 29.2°, but they can be found at even larger distances between the side aromatic rings of numerous peptides stabilizing their structure [41,42].

In order to predict the mechanical stability of the crystal of compound **1**, a avoid analysis was performed based on the isosurfaces of the procrystal electron density being the superposition of the spherical electron density at the corresponding nuclear position. The distribution of the electron density shows the void space in the crystal allows the calculation of the void surface and volume [43]. Figure S7 shows the void surface for compound **1**. The void volume in the unit cell is 120.97 Å, which indicates that the void percentage (9.85%) is relatively small and the degree of packing (0.901)—is relatively high. It indicates that crystal **1** can be subjected to significant stress without crushing and suggests its good mechanical properties.

### 2.4. Spectroscopic Studies

#### 2.4.1. FT–IR and Raman Spectra

In both the FT–IR Raman spectra of **1**, the medium and narrow bands at 3321 cm^−1^, 3203 cm^−1^, 3321 cm^−1^, and 3205 cm^−1^, respectively, are attributed to the N–H stretching vibrations (Figure S8). In the 3050–2929 cm^−1^ spectral range, the medium peaks in the Raman spectrum of complex **1** (in the brackets) are characteristic of ν(C–H) stretching vibrations. To distinguish the type of coordination of Cu(II) ions by the carboxyl group of the l–Br_2_Tyr ligand (between monodentate, chelating bidentate, and bridging bidentate) in **1**, it is sufficient to mark the difference in the frequency of the spectral bands originating from the antisymmetric stretching and the symmetric vibration of the COO^−^ group, expressed by the formula Δ = ν_as_(COO^−^) − ν_s_(COO^−^) [44]. In the FT–IR spectrum of **1**, the medium band found at 1611 cm^−1^ (1597 cm^−1^) can be assigned to the ν_as_(COO^−^), while ν_s_(COO^−^) coupled with ν(C−C)_L–Br2TyrRing_ stretching vibrations generate a strong band at 1428 cm^−1^ (1431 cm^−1^). The value of Δ = 183 cm^−1^ for **1** is greater than 110 cm^−1^, indicating the bidentate bridging coordination of the carboxylate group [44]. This is consistent with the crystal structure of **1**, which clearly shows that each oxygen bridging the carboxylate group bonds to different Cu(II) ions. The band at 1081 cm⁻^1^ in the FT-IR spectrum for **1** can be attributed to an ‘X-sensitive’ (X = Br in this case) mode “q” (C–Br in-plane deformation mode) as described by Whiffen [45]. This vibrational mode is influenced by the presence of the halogen substituent. The frequency of 1081 cm⁻¹ is higher than that observed for similar copper(II) complexes with L-tyrosine substituted for iodide instead of bromide, such as [Cu(phen)(L-I_2_TyrO^−^)(H_2_O)]·2H_2_O [19,20], which exhibits a band at 1057 cm⁻^1^ (coupled with ν(C–C). Additionally, ν(C–Br) is higher than ν(C–I) due to the lower reduced mass of bromine compared to iodine. The lattice vibrations and modes associated with the Cu(II) coordination sphere can be detected by analysis of the FIR (*far infrared*) region. The most interesting ν(Cu–L) stretching vibrations of the title complex and other Cu(II)–tyrosinato complexes are listed in Table 5 and shown in Figure S9.

It should be mentioned that the medium band at 425 cm^−1^ in the FT–IR and Raman spectra of **1** can be assigned to ν(Cu–N) stretching vibrations. The strong doublet at 336 cm^−1^ and 320 cm^−1^ is due to ν(Cu–O) stretching modes with the oxygen atom from the L–Br_2_Tyr. The splitting of this band results from the coordination of the Cu(II) center by O1 and O2i atoms from two different molecules of the ligand. The ν(Cu–Cl) stretching vibrations in complex **1** generate a medium-intensity band at 264 cm^−1^ in the IR spectrum [46]. The frequencies of Cu–L stretching vibrations derived from FT–IR and Raman spectra are collected in Table 5 for the series of Cu(II)-tyrosinato complexes. It transpires that they are generally greater when the ligand is l–Br_2_tyrosine.

#### 2.4.2. X- and Q-Band EPR Spectra

**Figure 6:**
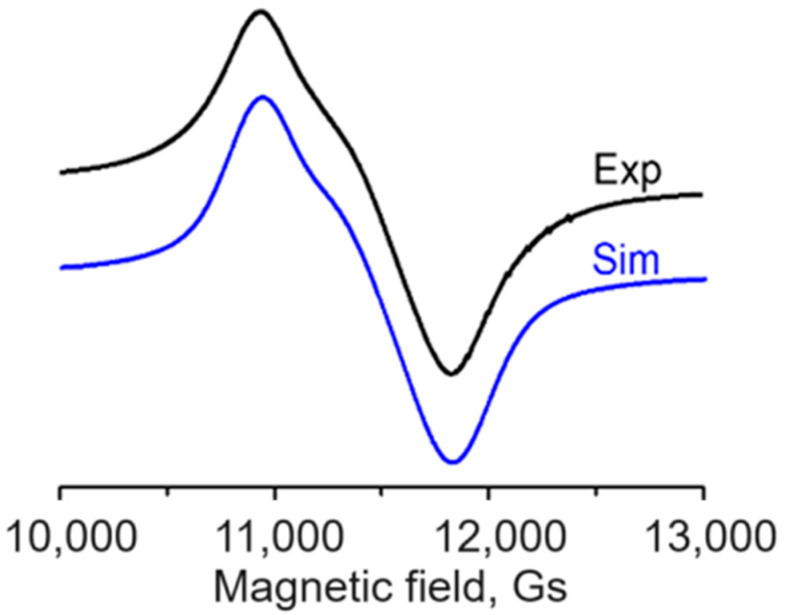
Q–band experimental EPR spectrum of polycrystalline complex **1** at 297 K, along with the spectrum simulated using the parameters given in the text.

The X-band spectra at 77K and 293K of the polycrystalline sample present a broad symmetrical line without any anisotropy due to the difference between the main components of the g tensor (Figure S10). This is in contradiction to the geometry of Cu(II) complexes, which always deviates from the regular, as predicted by the Jahn–Teller theorem. At a higher microwave frequency (Q–band ~ 36 GHz), the spectrum reveals a specific anisotropy due to the overlapping of three broad lines. Its computer simulation provided the best fit by using g_1_ = 2.218, g_2_ = 2.135, and g_3_ = 2.056 (Figure 6).

The crystal structure of **1** (see Section 2.1) revealed that two adjacent [CuCl(L-Br_2_Tyr)] complexes linked by a O–C–O bridge to form 1D chains are equivalent chemically but not crystallographically. It turns out that the base planes of these two Cu(II) complexes are inclined to each other at an angle of 2γ = 61.5° and rotated around the g_z_ axis by an angle of 2ξ = 39.7°. As a result, these differently oriented adjacent Cu(II) complexes undergo magnetic coupling over a relatively short distance (4.864 Å), and the computer simulation of the EPR spectral line shape provides cooperative (crystal) g parameters rather than molecular ones. Knowing the dependence of the known crystal parameters (g_1_, g_2_, and g_3_) on the unknown molecular parameters (g_z_, g_y_, and g_z_), the differences between them are trigonometric functions of the γ and ξ angles; thus, it is possible to calculate the diagonal components of the molecular g tensor [47]. The calculated values, g_z_ = 2.263, g_y_ = 2.095, and g_x_ = 2.049, correspond to the rhombic symmetry of the molecular g tensor, proving that the local symmetry of the Cu(II) coordination sphere is lowered from axial to rhombic. It is in agreement with the crystal and molecular structure. The symmetry of the six-coordinate polyhedron with Br and O (of OH group) at axial positions is lower than axial, as both the *cis* and *trans* angles sin the octahedron are significantly different from 90° and 180°, respectively (see Section 2.1). The value of the lowest parameter g_x_ greater than 2.04 and the relationship between the diagonal components of molecular g tensor g_z_ >> g_y_ > g_x_ > 2.04, corresponds to d_x2−y2_ orbital of the unpaired electron ground state [48]. The rhombic symmetry of the g tensor is consistent with elongated rhombic octahedral [49].

#### 2.4.3. NIR–Vis–UV Electronic Spectra

In the range from 9000 cm^−1^ to 21,500 cm^−1^, the diffuse–reflectance spectrum of the crushed crystals of compound **1** shows a broad and asymmetric single band originating from *d*–*d* type transitions for copper(II) center (d^9^ configuration) (Figure S11). Despite the geometrical distortion of the *cis*–[CuNO_2_Cl] chromophore, individual bands are not resolved clearly. A single broadband in the spectrum of **1**, like [CuO_4_] and [CuN_4_] complexes [50,51,52,53], could contain at least three *d-d* components. Indeed, the spectrum includes a shoulder around 11,500 cm^−1^ and three slightly marked maxima at ca. 15,800 cm^−1^, 16,800 cm^−1^, and 17,700 cm^−1^ (Figure S11). The true symmetry of the molecule is known to be C_i_ from the crystal structure determination. For the bulk of this discussion, the symmetry closer to the D_2h_ of the molecules is taken (deviation from square planar, the *trans*–N1^i^–Cu–O1, and O2–Cu–Cl1 bond angles are 164.5°, and 175.1°, respectively), and *d-d* transitions are forbidden by the Laporte rules but are invoked by the vibronic mechanism [54,55]. In the rhombic (D_2h_) crystal field, the degenerate spin allowed ^2^E_g_ (O_h_, ground state from ^2^D level) and ^2^T_2g_ (O_h_, excited state from ^2^D) terms to split into ^2^A_1g_(d_x2-y2_) + ^2^A_2g_(d_z2_) and ^2^B_1g_(d_xy_) + ^2^B_2g_(d_xz_) + ^2^B_3g_(d_yz_) states, respectively [56,57]. The shoulder and three maxima observed in the reflectance spectrum, therefore, suggest the following assignments: ^2^A_1g_(d_x2-y2_) → ^2^B_1g_(d_xy_) (11,500 cm^−1^), ^2^A_2g_(d_z2_) (15,800 cm^−1^), ^2^B_3g_(d_yz_) (16,800 cm^−1^), and ^2^B_2g_(d_xz_) (17,700 cm^−1^).

## 3. Materials and Methods

### 3.1. Materials

l–Br_2_Tyr monohydrate, CuCl_2_∙2H_2_O, HCl, methanol (MeOH), and KOH were obtained from Sigma. All chemicals used were of the highest grade available.

### 3.2. The Synthesis of {[CuCl(μ–O,O’–l–Br_2_Tyr)]}_n_ (**1**) 

0.107g l–Br_2_Tyr monohydrate was dissolved in a mixture consisting of 16 cm^3^ of MeOH and 4.0 cm^3^ aqueous solution of 0.2 M KOH. The 20 cm^3^ of this mixture was added dropwise under stirring into 20 cm^3^ of 0.015M aqueous solution of CuCl_2_∙2H_2_O (0.0514g). The l–Br_2_Tyr and copper(II) ions were in 1:1 stoichiometry. The final deep green solution was kept at room temperature for 7 days. The complex 1 formed blue crystals. These crystals are soluble in formamide (FORM), dimethylformamide (DMF), and dimethyl sulfoxide (DMSO), but are insoluble in water, methanol, acetonitrile, or acetone solutions. Characterization data: yield 62%, Calcd for chemical formula C_9_H_8_Br_2_ClCuNO_3_ (1) (FW = 436.97 g/mol) (%): C, 24.73; H, 1.85; Cl, 8.11; Cu, 14.54; Br, 36.57; N, 3.21; O, 10.98. The following was found: C, 24.62; H, 1.78; Cl, 8.05; N, 3.20. Elemental analysis of C, H, and N was carried out using the vario EL Cube analyzer from Elementar. The percentage of Cl was determined using the Schoeninger method (the Mikro K analyzer from Elementar, Langenselbold, Germany). 

### 3.3. Methods

#### 3.3.1. Single-Crystal and Powder X-ray Data Collection

The X-ray intensity data for crystal 1 was collected by means of graphite monochromatic MoKα radiation on a four-circle κ geometry KUMA KM–4 diffractometer (KUMA Diffraction Poland, Wrocław, Poland) with a two-dimensional area CCD detector. The ω-scan technique with Δω = 1.0° for each image was used for data collection. One image was employed as a standard after every 40 images for monitoring the crystal stability and data collection, and no correction on the relative intensity variations was necessary. Data collections were made at 100(1) K with the CrysAlis CCD software Version 1.171.33.42 [58]. Integration, scaling of the reflections, correction for Lorenz and polarization effects, and absorption corrections were performed by means of the CrysAlis Red program [58]. The structure was solved by the SHELXT direct methods [59] and refined with the SHELXL–2018 program [60]. The hydrogen atoms of OH and NH_2_ involved in the hydrogen bonds were located in the difference Fourier maps, and their xyz positions were refined with U_iso_ = 1.5U_eq_(O) and U_iso_ = 1.2U_eq_(N), while the H atoms joined to carbon atoms of the aromatic ring were refined as rigid with U_iso_(H) = 1.2U_eq_(C). The final difference Fourier maps showed no peaks of chemical significance. Details of the data collection parameters, crystallographic data, and final agreement parameters are shown in Table S1. The visualization of the structure was made using Diamond 3.0 software [61]. The purity of the obtained compound 1 was checked on a PANanalytical X’Pert powder diffractometer equipped with a Cu Kα radiation source (λ = 1.54182 Å). The diffraction data were recorded in the range of 5–45° at room temperature. The XRPD pattern measured for the synthesized sample was in good agreement with the XRPD patterns simulated from the respective single-crystal X-ray data (Figure S12), thus demonstrating that the crystal structures are truly representative of the bulk materials.

#### 3.3.2. Theoretical Calculations—QTAIM, NCI, and NBO

Bader’s quantum theory of atoms in molecules (QTAIM) [62] provided a closer look into the electron density distribution and traced bond critical points (BCPs) within the analyzed crystal. The complimentary noncovalent interaction index (NCI) method [63] was utilized for a qualitative investigation of weak interactions. Natural bond orbital (NBO) analysis provided a deeper insight into the strength of the interactions and clarified the nature of the chemical bonds inside the coordination sphere. The modeled structure, which underwent the above-mentioned analyses, consisted of two copper centers, bonded with chlorine atoms, and connected with three organic ligands (l–Br_2_Tyr), representing one polymer chain from the crystal. The structure also included an additional three l–Br_2_Tyr ligands from the neighboring chains needed for acquiring a precise image of the coordination sphere. The geometrical parameters were derived from crystallographic data, and only the hydrogen atom’s position was optimized. Computational studies were carried out at the PBE0-D3/d95v(d,p) [64,65,66] level of theory. For heavier atoms (Cu, Br, Cl) LanL2 pseudopotential was used combined with the LanL2DZ [66] basis set. Calculations were performed using Gaussian 16, Rev. C.01 [67] set of codes. QTAIM results were obtained with the AIMAll [68] suite of programs. MultiWFN [69] software (version 3.8.) was used to perform NCI analysis; the outcome was visualized using the VMD [70] program. Natural bond orbitals studies were conducted using NBO 6.0 [71], and graphical representations of the orbitals were prepared in Chemcraft [72].

#### 3.3.3. Hirshfeld Surface Analysis

Hirshfeld surface analysis (HAS) was used to examine short and long intermolecular interactions in the crystal of 1 by introducing its crystallographic information file (CIF) as an input file into Crystal Explorer version 21.5 [73]. HAS creates 3D molecular surface contours and 2D fingerprint plots which form a van der Waals (vdW) surface around the molecule and correspond to the space occupied by the molecule in the crystal. The vdW radii of the atoms served the purpose of determining and normalizing the contact from points on the HS to atoms inside (d_i_) and outside (d_e_) the surface [74]. It was possible to perform the Hirshfeld surface (HS) calculations by taking the whole molecule because of the polymeric structure of complex 1, and the asymmetric unit was used.

#### 3.3.4. Spectroscopic Methods (FT–IR, FT–Raman, X- and Q-Band EPR, NIR–VIS–UV)

ATR FT–IR spectra of the ligand, L–Br_2_Tyr and complex **1** were recorded with a BrukerVertex 70V vacuum (Bruker, Billerica, MA, USA) spectrometer equipped with a diamond ATR cell with a resolution of 2 cm^−1^ in the middle-infrared (4000–500 cm^−1^) and far-infrared (600–100 cm^–1^) regions at room temperature. The Raman spectrum of 1 was recorded on the Bruker dispersive Raman Spectrometer Senterra coupled with a confocal microscope with 532 nm and 633 nm lasers, lenses of different focal lengths, and sample stage with automatic shifts in the x, y, and z directions, which enable spectroscopic mapping of samples with the positioning accuracy of up to 0.0001 mm. The presented spectra were recorded in a range of 4450–45 cm^−1^, with a resolution of 9–18 cm^−1^, using a 532 nm exciting laser line and 25 × 1000 μm aperture. Additionally, the FT–Raman spectrum of l–Br_2_Tyr was collected on a Bruker MultiRam spectrometer (Bruker, Billerica, MA, USA) (Nd:YAG laser with a CW radiation at 1064 nm) equipped with a liquid N_2_ cooled germanium detector at a resolution of 4 cm^−1^, co–addition of 1024 scans, and laser power values of 500 mW. The spectral data were collected and further elaborated using Bruker OPUS 7.5 software. The EPR spectra of polycrystalline compound 1 were recorded by means of a Bruker Elexsys E500 spectrometer (Bruker, Billerica, MA, USA) equipped with an NMR teslameter and a frequency counter. The X-band spectra were recorded at 298 and 77 K, while the Q-band spectra were recorded at 298 K. The simulations of the experimental spectra were performed using a computer program DoubletExact (S = 1/2), employing full diagonalization of the spin Hamiltonian matrix [75], written by Prof. A. Ozarowski (National High Magnetic Field Laboratory, Florida, University, Tallahassee, FL, USA). The diffuse–reflectance NIR–Vis–UV electronic spectra were obtained on a Cary 500 Scan spectrophotometer over the range of 5000–50,000 cm^−1^ with a measured step of 10 cm^−1^ at 293 K for complex **1** and l–Br_2_Tyr. These spectra were measured with identical parameters as a baseline of the white reference sample.

## 4. Conclusions

Coordination polymers based on the l–Br_2_Tyr ligand (with a protonated OH group despite alkaline synthesis conditions) are relatively rare. The structure of titled 1D Cu(II) polymer (**1**) consists of [CuCl(l–Br_2_Tyr)] monomers bridged by a single carboxylate group of the ligand in a bidentate *syn*–*anti* bonding mode*,* with a Cu···Cu distance of 4.864 Å. This bridging mode is also manifested by the value Δ = 183 cm^−1^ and the crystal type (not molecular type) values of g parameters: g_1_ = 2.218, g_2_ = 2.135, and g_3_ = 2.056. These values correspond to the averaged diagonal components of g tensors of two bridged and differently oriented [CuCl(l–Br_2_Tyr)] monomers, with the base planes inclined by 61.5° to each other and rotated by 39.7° around the g_z_ axis. The Cu(II) center in each monomer is equatorially coordinated by the independent Cl1 ion, the chelating N and O atoms of the parent ligand, and the O atom of the symmetry-related ligand of the adjacent monomer. Neighboring 1D polymer chains with coordination interact within the 3D structure through the weak intermolecular contacts (at distances of about 3 Å) between the Cu(II) center in one chain and the O atom (from the OH group) and the Br atom of the ligands in the neighboring chains. The use of quantum chemical calculations (NCI and NBO) and Hirshfeld structure analysis allowed the theoretical verification of the intermolecular contacts originating from adjacent chains. The symmetry of the resulting octahedral geometry of the Cu(II) coordination sphere is lower than the axial one due to angle and distance deviations, which is confirmed by the rhombic symmetry of the molecular tensor g (g_z_ = 2.263, g_y_ = 2.095, g_x_ = 2.049) calculated on the basis of the crystal EPR parameters g. The values and the relationship between the diagonal molecular g tensor components correspond to the d_x2-y2_ orbital of the unpaired electron ground state. Taking these observations into account the transitions of the *d-d* band components appearing in the electron reflection spectrum were assigned ^2^A_1g_(d_x2-y2_) → ^2^B_1g_(d_xy_) (11,500 cm^−1^), ^2^A_2g_(d_z2_) (15,800 cm^−1^), ^2^B_3g_(d_yz_) (16,800 cm^−1^), and ^2^B_2g_(d_xz_) (17,700 cm^−1^). The three-dimensional supramolecular architecture of the studied complex results from the system of hydrogen bonds O–H···O, N–H···Br, N–H···Cl, and the stabilizing Cg···Br, Cu···O, and Cu···Br interactions. Hirshfeld surface analysis showed the dominant contribution of the H···Br, H···O, and H···Cl contacts to the stabilization of the crystal packing compared to other contacts.

## 5. Patents

A. Wojciechowska, J. Janczak, Patent. Poland, no. PL 241662, publ. 14 November 2022. Crystalline form of the poly{(*μ–*dibromo-L-tyrosine O,O’)chlorocopper(II)} complex and its preparation method/Wrocław University of Science and Technology, Wrocław, Poland.

## Figures and Tables

**Table 1:** Selected interatomic distances (Å) and bond angles (°) for the compound **1**.

Bond Lengths (Å)				Bond Angles (°)	
Cu–O1	1.944(4)	Cu–O2^i^	1.981(4)	O1–Cu–N1^i^	164.46(19)
Cu–N1^i^	1.972(4)	Cu–Cl1	2.2217(15)	O1–Cu–O2^i^	85.81(17)
O1–C1	1.242(7)	O2–C1	1.276(6)	N1^i^–Cu–O2^i^	82.68(17)
C6–Br1	1.907(5)	C2–N1	1.482(7)	O1–Cu–Cl1	96.80(13)
C8–Br2	1.896(5)			N1^i^–Cu–Cl1	95.46(14)
				O2^i^–Cu–Cl1	175.11(12)

Symmetry codes: (i) *x* − 1/2, −*y* + 1/2, −*z* + 1.

**Table 2:** Proposed hydrogen bonds, contacts, and intramolecular interactions (in Å) for **1**.

D–H···A	D–H	H···A	D···A	D–H···A	I(J)	Me	I···Me	Cg(I)	Cg(J)	Cg···Cg	Cg(I)	I(J)	Cg···I
O3–H3···O2^iii^	0.88	1.98	2.804 (5)	151	O3^vi^	Cu	3.131(8)	*Cg*(1)	*Cg*(1)^iv^	5.798(3)	*Cg*(1)	Br2^iv^	3.450(2)
N1–H1*A*···Br2^iv^	0.81	2.93	3.651 (4)	149	Br1^vii^	Cu	3.168(8)	*Cg*(1)	*Cg*(1)^viii^	5.161(3)			
N1–H1*B*···Cl1^v^	0.87	2.42	3.280 (5)	171									

Symmetry codes: (iii) −*x* + 3/2, −*y* + 1, *z* + 1/2; (iv) −*x* + 2, *y* − 1/2, −*z* + 3/2; (v) *x*, *y* + 1, *z*; (vi) −*x* + 3/2, −*y* + 1, *z* − 1/2; (vii) −*x* + 1, *y* − 1/2, −*z* + 3/2; (viii) *x* + 1, *y* + 1/2, −*z* + 3/2. *Cg*(1) is centroid of C4–C5–C6–C7–C8–C9 ring.

**Table 3:** AIM parameters. Bond critical point (BCP) properties: electron density *ρ*, Laplacian of the electron density ∇^2^*ρ*, total electron energy H, kinetic energy (G), and potential energy (V). All values in a.u.

	*ρ*	∇^2^*ρ*	H	G	V
Cu–N	0.092	+0.443	−0.016	+0.126	−0.142
Cu–O1	0.086	+0.512	−0.008	+0.136	−0.144
Cu–O2	0.076	+0.498	−0.003	+0.128	−0.131
Cu–Cl	0.069	+0.281	−0.004	+0.074	−0.078
Cu···Br	0.014	+0.027	−0.002	+0.008	−0.010

**Table 4:** NBO results. Dominant donor–acceptor interaction with the copper center within the first coordination sphere. Sum of second-order energy (E^2^) values in kcal/mol.

	Donor → Acceptor	ΣE^2^
O2–Cu	LP (O) → σ* (Cu-Cl) (70) ^a^	72.52
O1–Cu	LP (O) → LV (Cu) (86)	69.41
N–Cu	LP (N) → LV (Cu) (84)	62.89
Br···Cu	LP (Br) → LV (Cu) (79)	10.65
HO···Cu	LP (O) → LV (Cu) (67)	1.16

^a^ percentage of dominant interaction; σ*(Cu-Cl) denotes antibonding Cu-Cl sigma orbital.

**Table 5:** The comparison of the selected ν(Cu–L) stretching modes in FIR and Raman spectra of the Cu(II)–L–X_2_Tyr complexes synthesized by our group; frequencies in cm^−1^.

	1		[CuCl(L–I_2_Tyr) (phen)]∙2H_2_O [20]		[Cu(L–I_2_Tyr)(H_2_O) (phen)]∙(NO_3_) [20]		[Cu(L–I_2_TyrO^−^)(H_2_O) (phen)]∙2H_2_O [19]	
modes	FIR	Raman	FIR	Raman	FIR	Raman	FIR	Raman
ν(Cu–N_L-X2Tyr_)	425 m	425 m	431 m	433 s	433 m	434 s	425 m	432 m
ν(Cu–O_L-X2Tyr_)	336/320 s	323 w	353 m	361 vw	358/349 m	362 vvw	350 m	354 vw
ν(Cu–Cl) *	264 m	263 m	ov	ov	−	−	−	−

X = I or Br atoms; *—Regular below 300 cm^−1^ coupled with lattice vibrations; abbreviations: s—strong, m—medium, w—weak, v—very, and ov—overlapped.

## Data Availability

Data are contained within the article and Supplementary Materials.

## Supplementary Materials

The following supporting information can be downloaded at: https://www.mdpi.com/article/10.3390/molecules29112709/s1, Table S1: Summary of the crystallographic data for complex **1**. Figure S1. (a) The coordination mode for l–Br_2_Tyr in 1 with labeling scheme and (b) coordination environment of the Cu(II) center. Displacement ellipsoids are drawn at a 50% probability level, except for the H atoms which are drawn as a solid ellipse with arbitrary radii. (picture a). Symmetry codes: (i) *x* − 1/2, −*y* + 1/2, −*z* + 1, (ii) *x* + 1/2, −*y* + 1/2, −*z* + 1. Figure S2: The stabilization role of π···π interactions in connection with neighboring 1D coordination chains and the *Cg*···*Cg* distances between the nearest aromatic rings of l–Br_2_TyrOH ligands. All C-bounded H-atoms are omitted for clarity. Figure S3. The 3D supramolecular structure. Table S2: Detailed NBO results, the second-order energy values (E^2^) of interacting orbitals in kcal mol^−1^. Figure S4. View of interacting NBO localized orbitals within the coordination sphere: (a) O···Cu and (b) Br···Cu. Model fragment of {[CuCl(*μ–O,O’*–l–Br_2_Tyr)]}_n_ complex used for calculations; LP filled with lone-pair orbitals and LV are lone vacancy type orbitals (unfilled valence nonbonding orbitals). Table S3. The percentage of actual contacts, random contacts, surface percentage on the molecular structure for a particular atom, and enrichment ratios for the pair of atoms involved in intermolecular interactions for 1 with random contact greater than 0.9%. Figure S5. Vital fingerprint plots of 1, showing the contributions of a particular pair of atoms involved in intermolecular contacts indicated by blue areas. For every fingerprint plot, the gray area represents the integrity plot. Surface maps below each fingerprint plot indicate the blue regions that are associated with these specific intermolecular contacts. Figure S6: Decomposed fingerprint plots for 1 show the shares of contacts between specific pairs of atoms to total HS, represented as a blue area; the gray shadow outlines the full fingerprint. On the surface below, the share of that particular contact is shown as a blue region. Figure S7: Voids surface for the unit cell based on 0.0002 a.u isosurfa. Figure S8: (a) FT–IR spectra of complex **1** and l–Br_2_Tyr; (b) Raman spectra of complex **1**; and (c) Raman spectrum of puree ligand (l–Br_2_Tyr). Figure S9. The comparison of spectra of complex **1** and other copper(II) L–I_2_tyrosinato compounds in the range of 600–50 cm^−1^: (a) FT–FIR spectra and (b) FT–Raman spectra. Figure S10: Polycrystalline X-band EPR spectrum of 1 at 273 K. Figure S11: The diffuse–reflectance spectra of solid samples of 1 and l–Br_2_TyrOH. Figure S12: (a) Simulated from single crystal and (b) experimental XRPD pattern. Scheme S1. Scheme of the fragment of {[CuCl(*μ–O,O’*–l–Br_2_Tyr)]}_n_ polymer structure cut out from crystal lattice and used in theoretical calculations (AIM, NCI, and NBO). CCDC number 2340395 contains the supplementary crystallographic data for the complex {[CuCl(*μ–O, O’*–l–Br_2_Tyr)]}_n_ (**1**).
