# Supplementary material for: Unique Use of Dibromo–L–Tyrosine Ligand in Building of Cu(II) Coordination Polymer—Experimental and Theoretical Investigations
Source: Molecules. 2024 Jun 6;29(11):2709. doi: 10.3390/molecules29112709 (PMC11173859; doi:10.3390/molecules29112709)
Supplement: Supplementary file 1 [file molecules-29-02709-s001.zip › molecules-3014012-supplementary.pdf]

Highlights

♦ A new copper(II) coordination polymer based on L–Br_2TyrOH moieties was synthesized in the form of a crystal in alkaline solution

♦ The L–Br_2TyrOH carboxylate group bridges Cu(II) monomeric units in a *syn–anti* bidentate mode with the Cu···Cu^{ii} separation of 4.864 Å

♦ The sphere around Cu(II) in one chain is completed by Br and phenolic O atoms originating from ligands in adjacent chains

Supplementary Information

Unique use of dibromo–L–tyrosine ligand in building of Cu(II) coordination polymer – experimental and theoretical investigations

Agnieszka Wojciechowska[a*], Jan Janczak[b], Tomasz Rojek[a], Muhammad Ashfaq[c], Magdalena Malik[a], Natasza Trzęsowska [a], Rafał Wysokiński[a] and Julia Jezierska[d]

[a] Faculty of Chemistry, Wrocław University of Science and Technology, Wybrzeże Wyspiańskiego 27, 50–370 Wrocław, Poland

[b] Institute of Low Temperature and Structure Research Polish Academy of Sciences, Okólna 2, 50–422 Wrocław, Poland,

[c] Department of Physics, University of Sargodha, Punjab, Islamic Republic of Pakistan,

[d] Faculty of Chemistry, University of Wrocław, Joliot–Curie 14, 50–383 Wrocław, Poland,

AUTHOR EMAIL ADDRESS agnieszka.wojciechowska@pwr.edu.pl

Corresponding Author. Agnieszka Wojciechowska, Faculty of Chemistry, Wrocław University of Science and Technology, Wybrzeże Wyspiańskiego 27, 50–370 Wrocław, Poland Phone: +48 713202280.

Table S1. Summary of the crystallographic data for the complex **1**.

Compound	**1**
CCDC No.	
Chemical formula	$C_9H_8Br_2ClCuNO_3$
M_r	436.97
Crystal system	Orthorombic
Space group	$P2_12_12_1$
a (Å)	7.3622(2)
b (Å)	8.0413(3)
c (Å)	20.7445(8)
α (°),β (°), γ (°)	
V (Å^3)	1228.11(7)
T (K)	100
Z	4
D_c(g·cm^{-3})	2.363
μ (mm^{-1})	8.50
Crystal size (mm)	0.21×0.16×0.12
Diffractometer	KUMA KM–4 with CCD detector
Temperature,	100(1)
Radiation type	Mo $K\alpha$
Wavelenght (Å)	0.71073
θ range(°)	2.717÷ 27.993
Absorption, T_{min}./T_{max}.	Multi–scan, 0.7893/1.0
F(000)	836
Refl. measured	18887
Refl. independent	2952
Refl. observed [I> 2σ(I)]	2725
No. of parameters	163
No. of restrains	1
R_{int}	0.047
R[F^2> 2σ(F^2)]	0.027
wR(F^2)	0.057
S	1.03
Absolute structure parameter	−0.009 (6)
$\Delta\varrho_{max}$, $\Delta\varrho_{min}$ (e Å^{-3})	0.49, -0.71

$wR=\{\Sigma [w(F_o^2–F_c^2)^2]/\Sigma wF_o^4\}^{½}$; $w^{-1}=\sigma^2(F_o^2) + (aP)^2 + bP$ where $P = (F_o^2 + 2F_c^2)/3$.
The a and b parameters are 0.0224 and 1.8412.

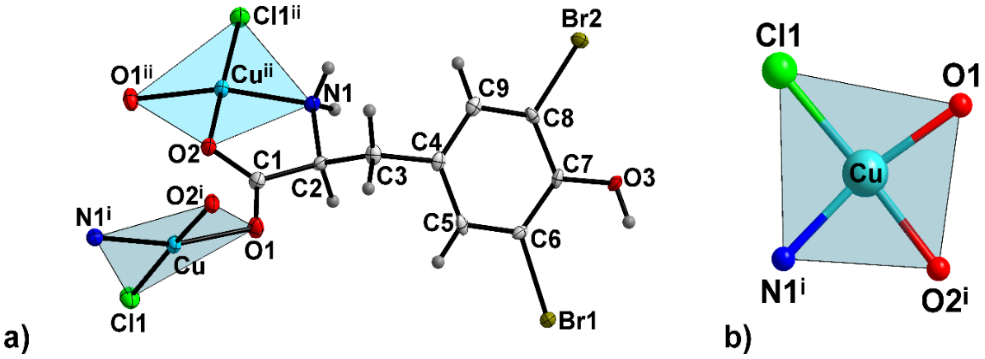

Figure S1. (a) The coordination mode for L−Br2Tyr in **1** with labelling scheme and (b) coordination environment of the Cu(II) center. Displacement ellipsoids are drawn at 50% probability level except for the H atoms which are drawn as a solid ellipse with arbitrary radii. (picture a). Symmetry codes: (i) *x*−1/2, −*y*+1/2, −*z*+1, (ii) *x*+1/2, −*y*+1/2, −*z*+1.

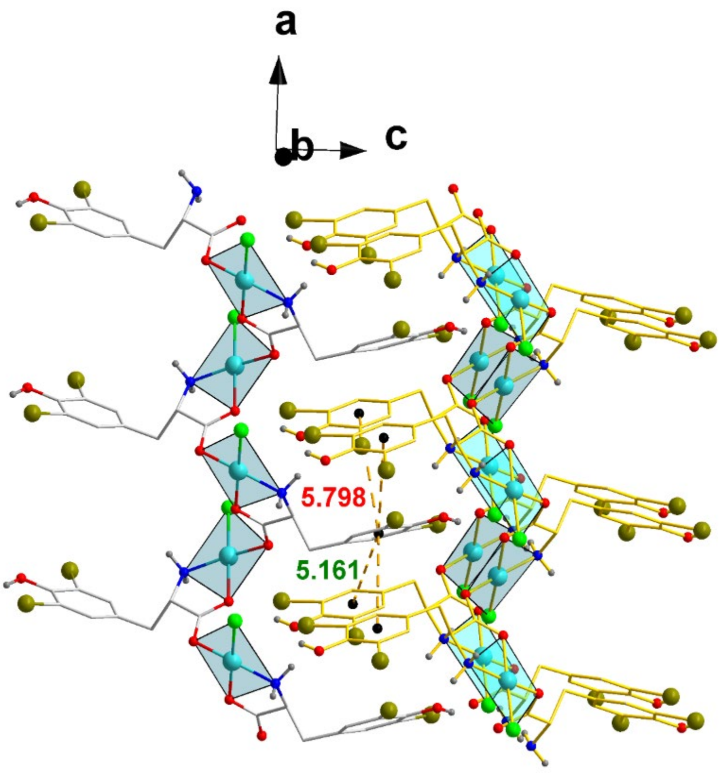

Figure S2. The stabilization role of π···π interactions in connection with neighboring 1D coordination chains and the *Cg*···*Cg* distances between the nearest aromatic rings of L–Br2Tyr ligands. All C–bounded H–atoms are omitted for clarity.

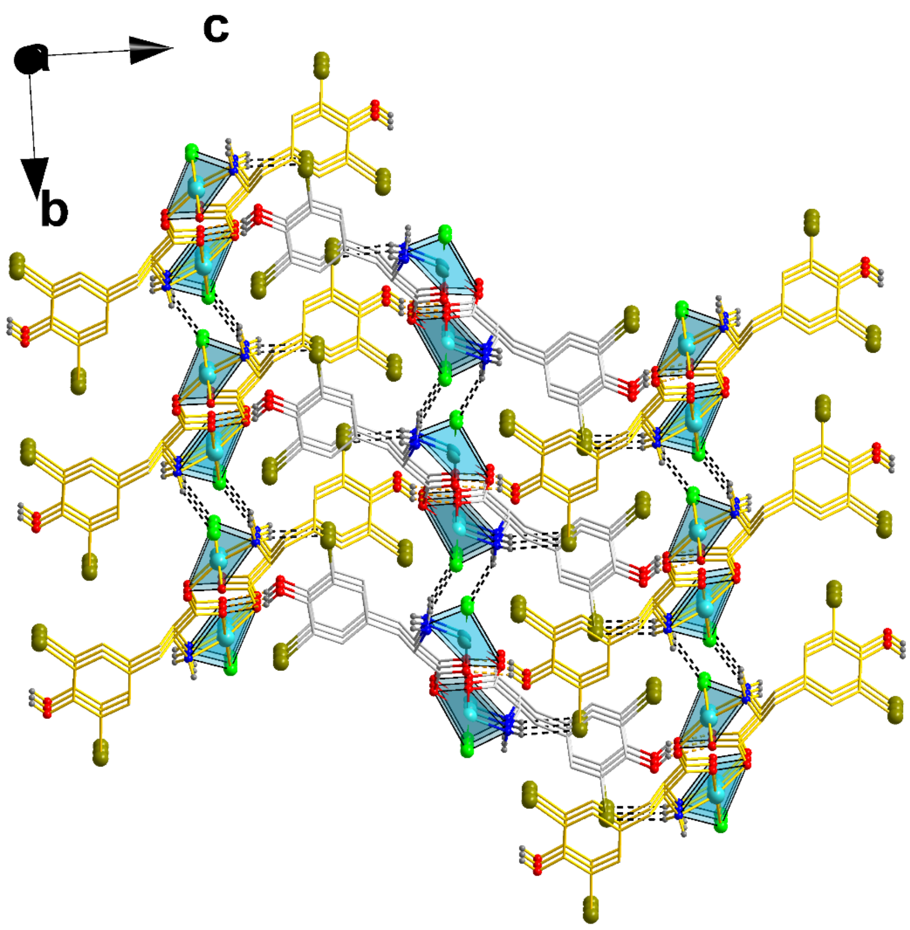

Figure S3. The 3D supramolecular structure of **1**. The O–H···O hydrogen bonds are shown as light orange dashed lines and weak N–H···Cl, N–H···Br interactions are shown as black dashed lines. All C−bounded H−atoms are omitted for clarity.

Table S2. Detailed NBO results, the second-order energies values (E^2) of interacting orbitals in kcal mol^{-1}.

	donor → acceptor	E^2
Br ··· Cu	LP (Br) → LV (Cu)	8.43 (79.2%)
	LP (Br) → σ* (Cu-Cl)	2.22 (20.8%)
	Σ	10.65
HO···Cu	LP (O) → LV (Cu)	0.78 (67.2%)
	σ (O-H) → LV (Cu)	0.18 (15.5%)
	LP (O) → σ* (Cu-Cl)	0.20 (17.2%)
	Σ	1.16
O1–Cu	LP (O) → LV (Cu)	59.38 (85.5%)
	LP (O) → σ* (Cu-Cl)	9.04 (13.0%)
	σ (C-O) → Cu	0.99 (1.4%)
	Σ	69.41
O2–Cu	LP (O) → LV (Cu)	21.23 (29.3%)
	LP (O) → σ* (Cu-Cl)	50.38 (69.5%)
	σ (C-O) → Cu	0.65 (0.9%)
	σ (C-O) → σ* (Cu-Cl)	0.26 (0.4%)
	Σ	72.52
N–Cu	LP (N) → LV (Cu)	53.01 (84.3%)
	LP (N) → σ* (Cu-Cl)	4.63 (7.4%)
	σ (C-N) → LV (Cu)	1.10 (1.7%)
	σ (C-N) → σ* (Cu-Cl)	0.05 (0.1%)
	σ (N-H) → LV (Cu)	4.05 (6.4%)
	σ (N-H) → σ* (Cu-Cl)	0.05 (0.1%)
	Σ	62.89
Cl–Cu	σ Cl – Cu<br>$d^{9.20}$<br>s(9.79%) p(0.14%) d(90.07%)	
	LP (Cl) → LV (Cu)	11.77 (79.5%)
	LP (Cl) -> σ* (Cu-Cl)	1.64 (11.1%)
	σ (Cu-Cl) → σ* (Cu-Cl)	1.40 (9.5%)
	Σ	14.81

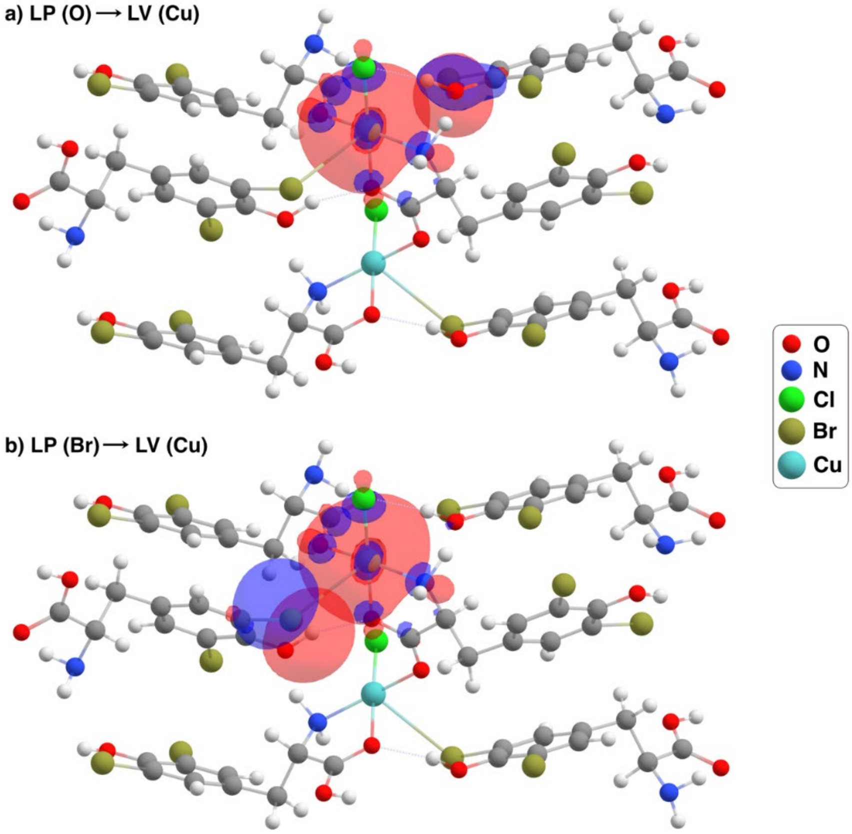

Figure S4. View of interacting NBO localized orbitals within coordination sphere (a) O···Cu and (b) Br···Cu. Model fragment of {[CuCl(*μ*–*O,O′*–L–Br_2Tyr)]}$_n$ complex used for calculations; LP are filled with lone-pair orbitals and LV are lone vacancy type orbitals (unfilled valence nonbonding orbitals).

Table S3. The percentage of actual contacts, random contacts, surface percentage on the molecular structure for particular atom and enrichment ratios for the pair of atoms involved in intermolecular interactions for **1** with random contact greater than 0.9%.

	**Atom**	**H**	**C**	**N**	**O**	**Br**	**Cl**	**Cu**
Contact %	**H**	3.7	9.5		17.5	22.9	17	3.1
	**C**	9.5	0.7		1.6	7.4	0.3	
	**N**				0.2			2.4
	**O**	17.5	1.6	0.2	1.5		0.3	4
	**Br**	22.9	7.4			1.4	3.5	2.3
	**Cl**	17	0.3		0.3	3.5	0.7	
	**Cu**	3.1		2.4	4	2.3		
Surface%		38.7	10.1	1.3	13.3	19.45	11.25	5.9
Random Contacts %	**Atom**	**H**	**C**	**N**	**O**	**Br**	**Cl**	**Cu**
	**H**	14.98						
	**C**	7.82	1.02					
	**N**	1.01	0.26	0.02				
	**O**	10.29	2.69	0.35	1.77			
	**Br**	15.05	3.93	0.51	5.17	3.78		
	**Cl**	8.71	2.27	0.29	2.99	4.38	1.27	
	**Cu**	4.57	1.19	0.15	1.57	2.30	1.33	0.35
Enrichment ratio	**Atom**	**H**	**C**	**N**	**O**	**Br**	**Cl**	**Cu**
	**H**	0.25						
	**C**	1.22	0.69					
	**N**	0.00						
	**O**	1.70	0.60		0.85			
	**Br**	1.52	1.88		0.00	0.37		
	**Cl**	1.95	0.13		0.10	0.80	0.55	
	**Cu**	0.68	0.00		2.55	1.00	0.00	

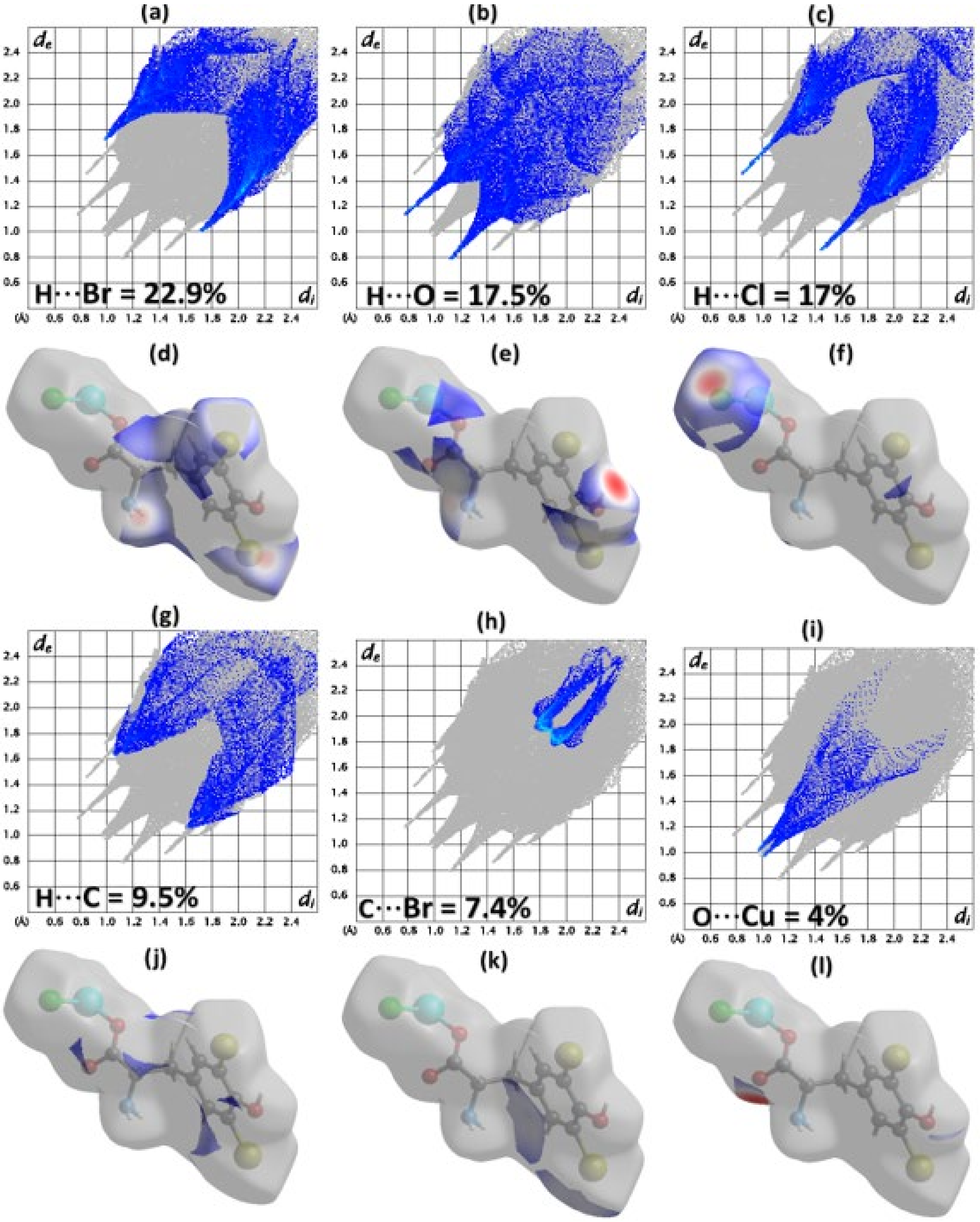

Figure S5. Vital fingerprint plots of **1**, showing the contributions of particular pair of atoms involved in intermolecular contacts.as blue areas. For every Fingerprint plot, the grey area represents the integrity plot. Surface maps below each fingerprint plot indicate the blue regions that are associated with these specific intermolecular contacts.

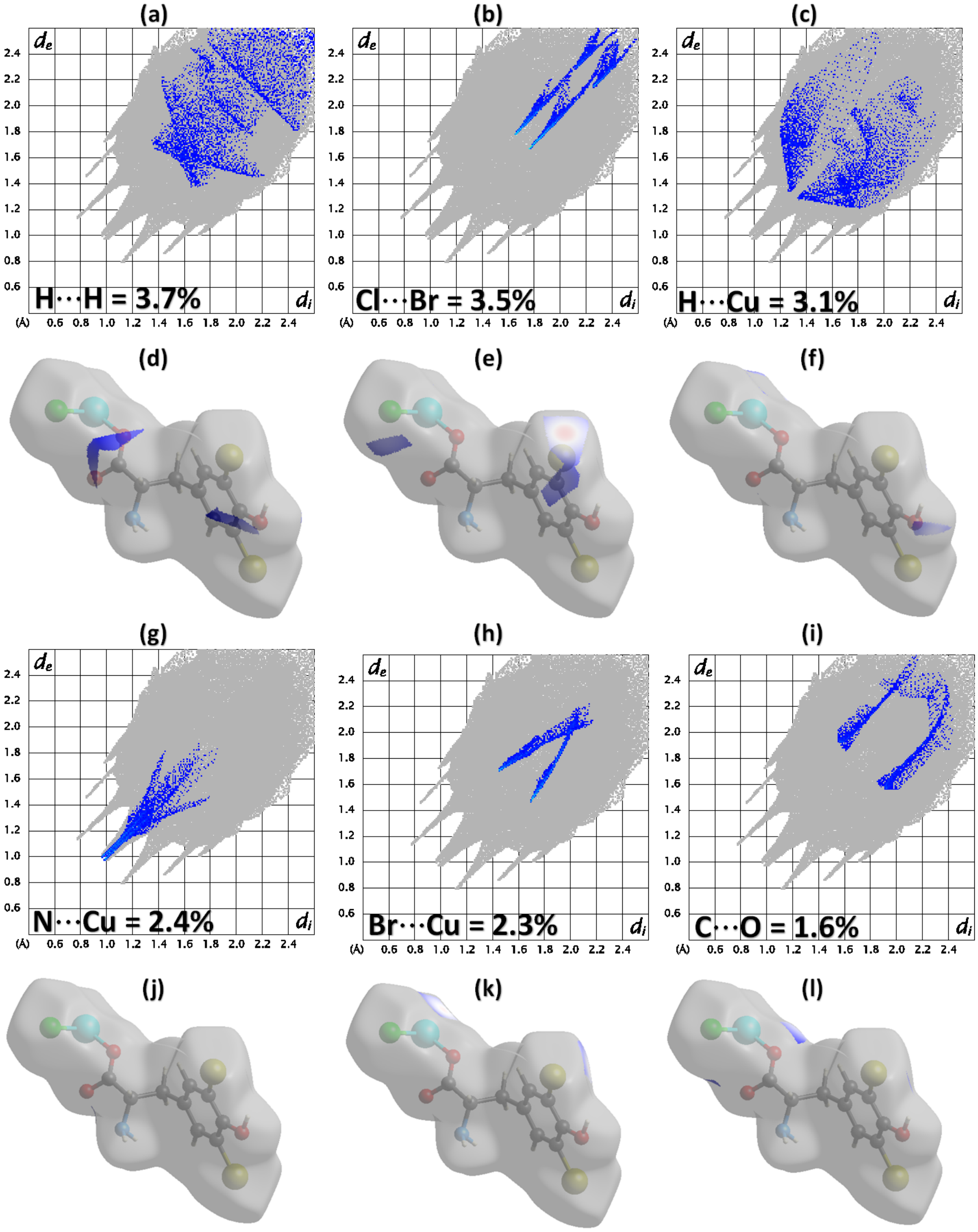

Figure S6. Decomposed fingerprint plots for **1** showing the shares of contacts between specific pairs of atoms to total HS as a blue area; the gray shadow outlines the full fingerprint. On the surface below, the share of that particular contact is shown as a blue region.

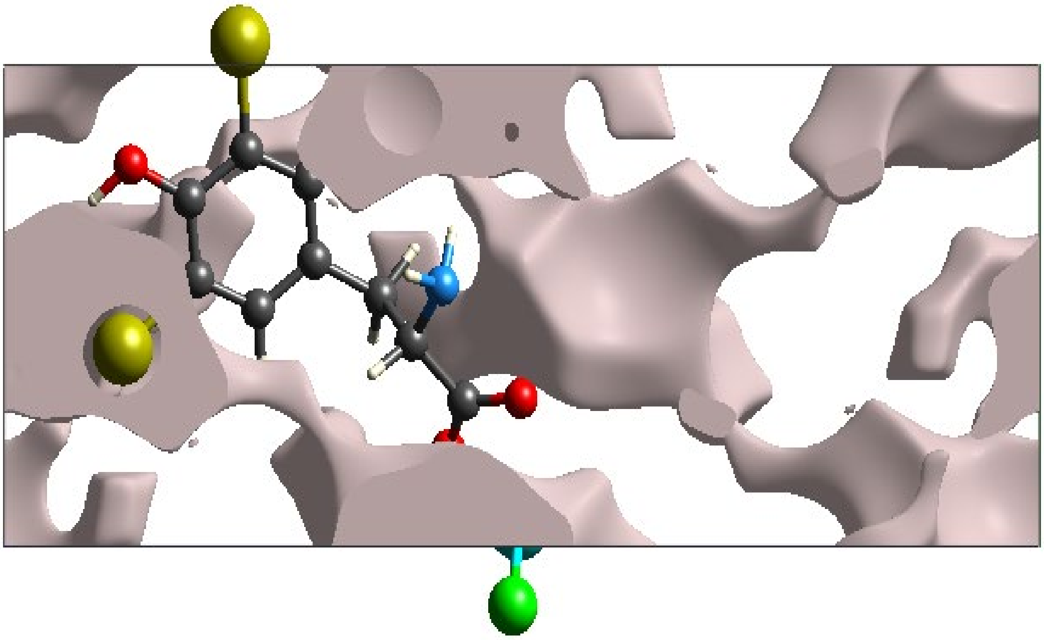

Figure S7. Voids surface for unit cell of based on 0.0002 a.u isosurface.

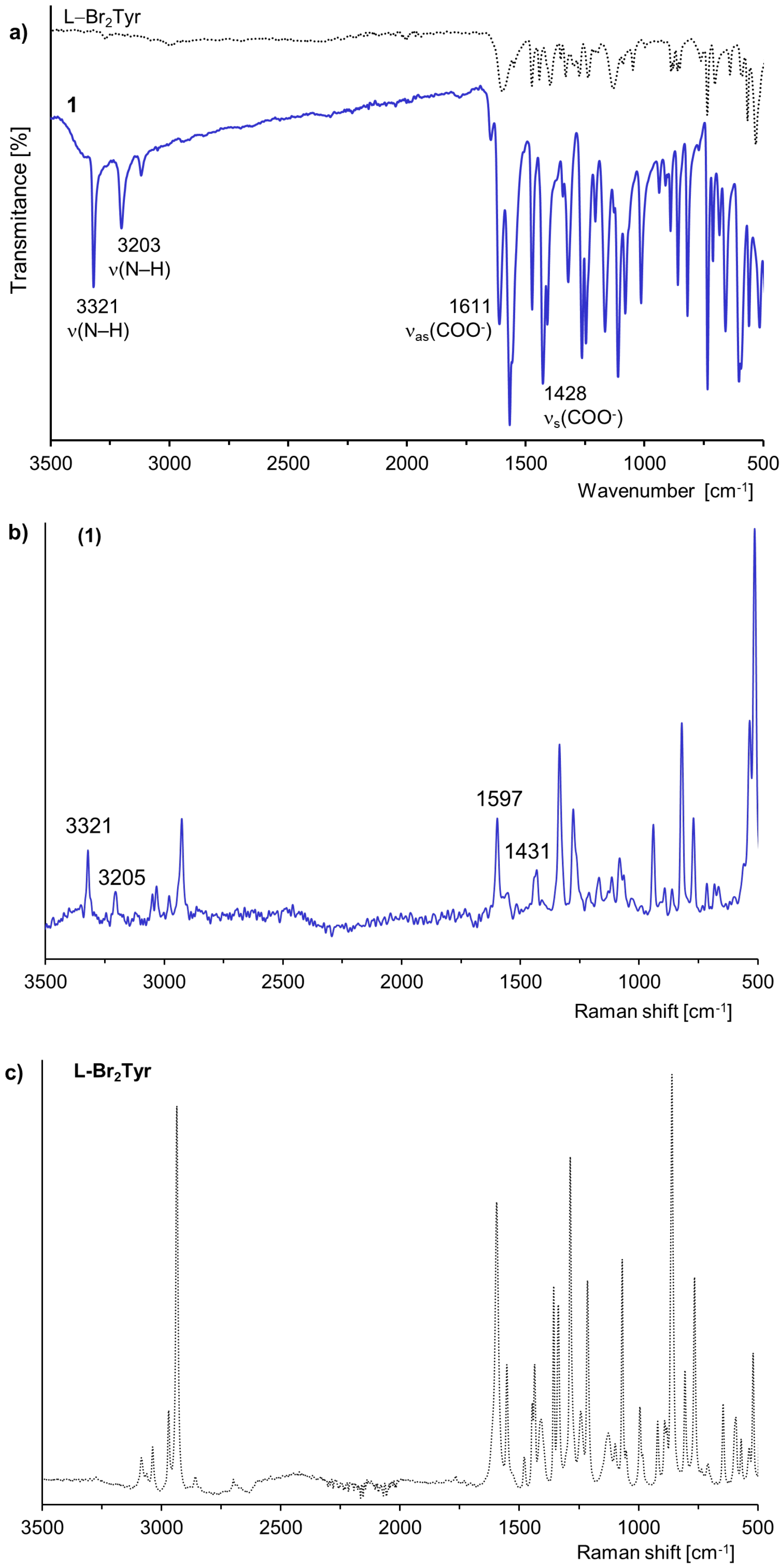

Figure S8. (a) FT–IR spectra of complex **1** and L–Br2Tyr; (b) Raman spectra of complex **1** and (c) Raman spectrum of puree ligand (L–Br2Tyr).

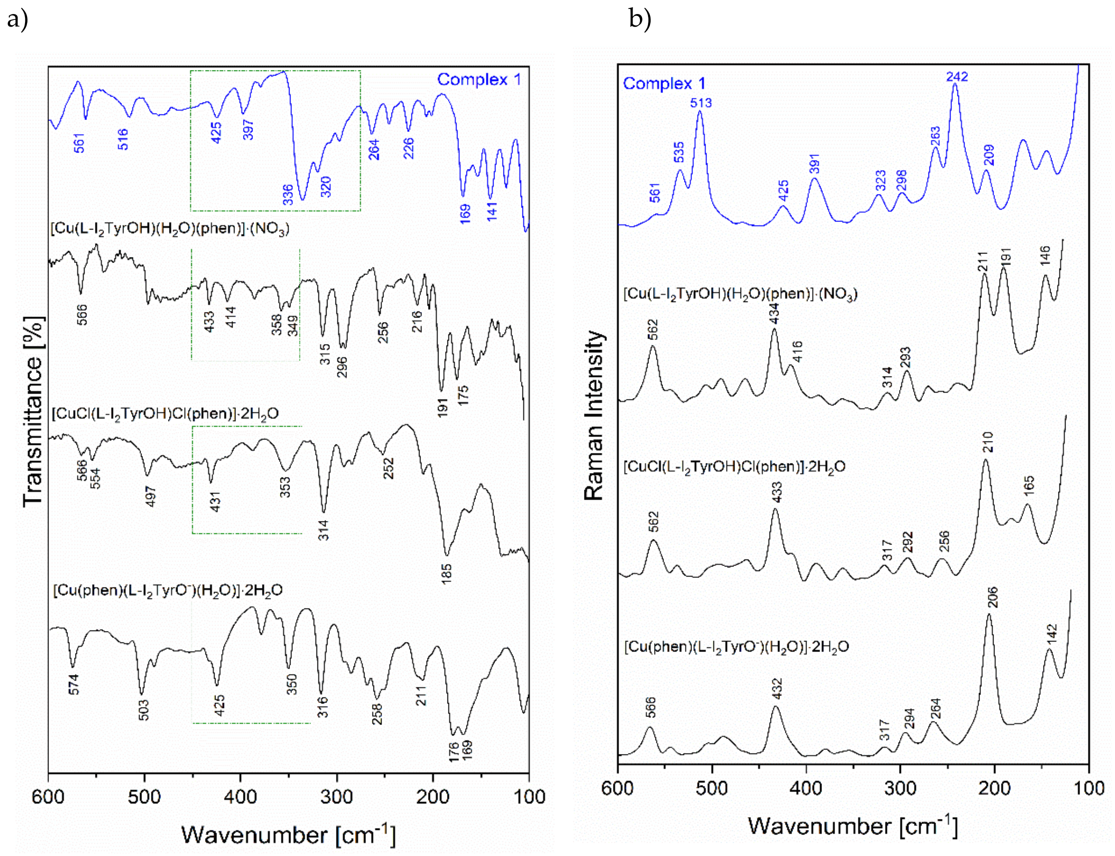

Figure S9. The comparison of spectra of complex **1** and other copper(II) L–I2tyrosinato compounds in the range 600–50 cm-1 (a) FT–FIR spectra and (b) FT–Raman spectra.

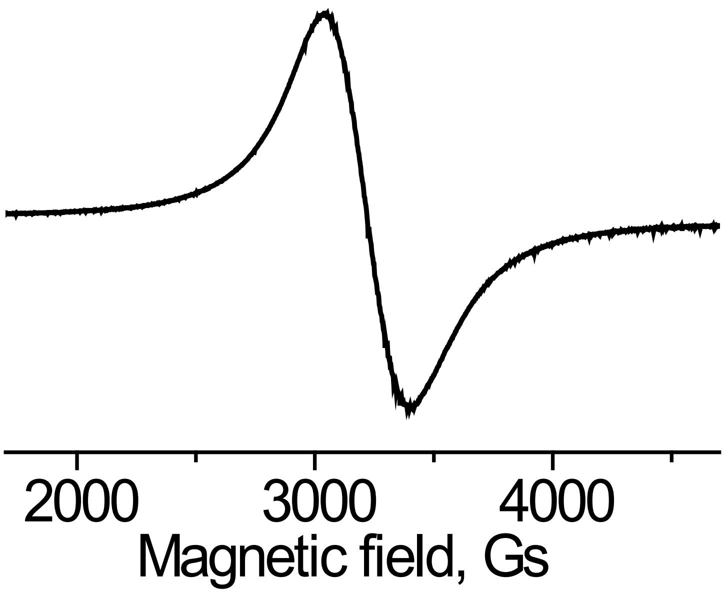

Figure S10. Polycrystalline X–band EPR spectrum of **1** at 273 K.

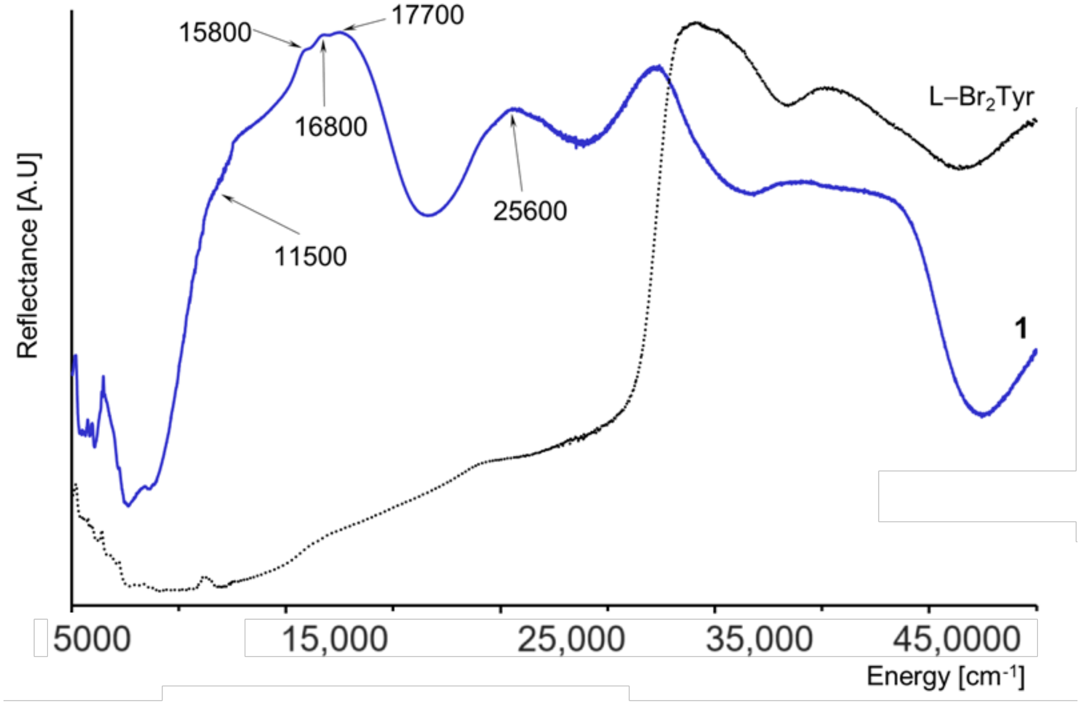

Figure S11. The diffuse–reflectance spectra of solid samples of **1** and L–Br_2Tyr.

(a)

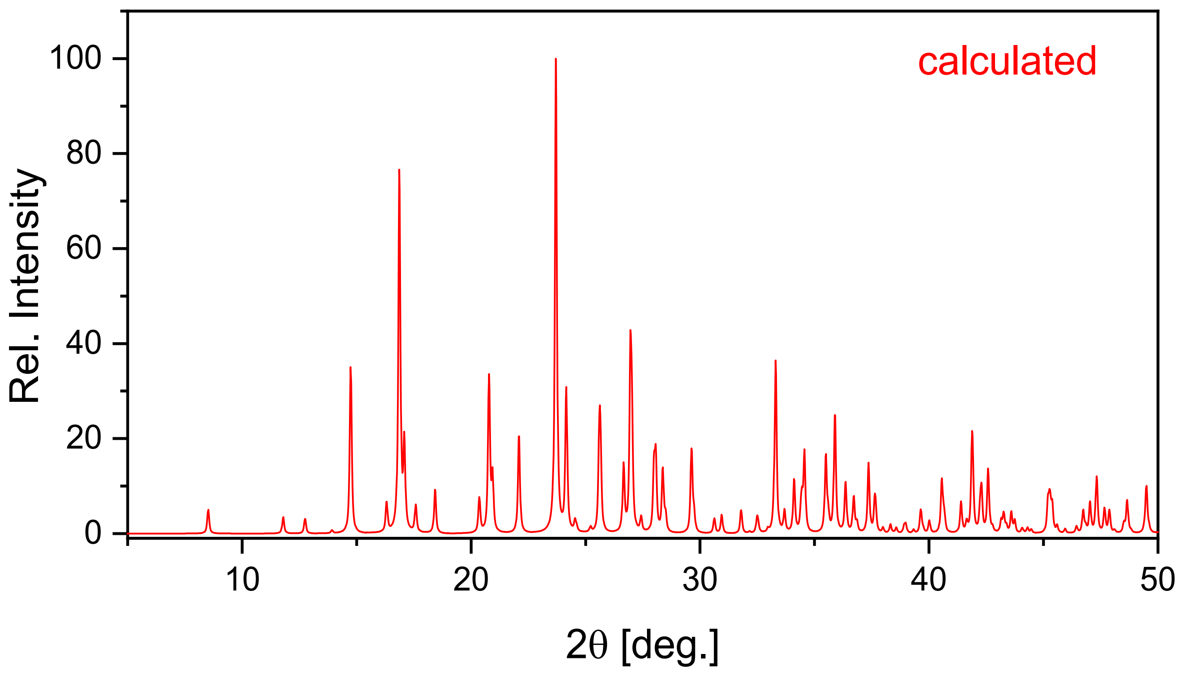

(b)

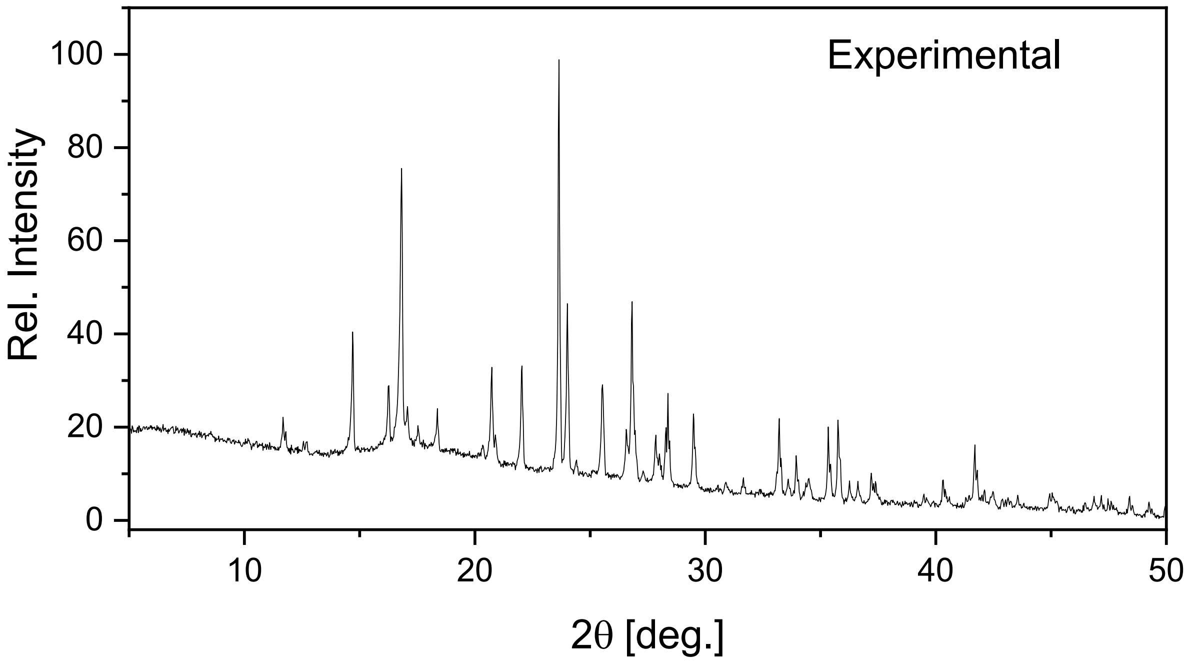

Figure S12. (a) Simulated from single crystal and (b) experimental XRPD pattern. To confirm the phase purity of the synthesized complexes, the PXRD pattern of compound **1** was recorded. The XRPD pattern measured for the synthesized sample was in good agreement with the XRPD patterns simulated from the

respective single–crystal X–ray data, thus demonstrating that the crystal structures are truly representative of the bulk materials.

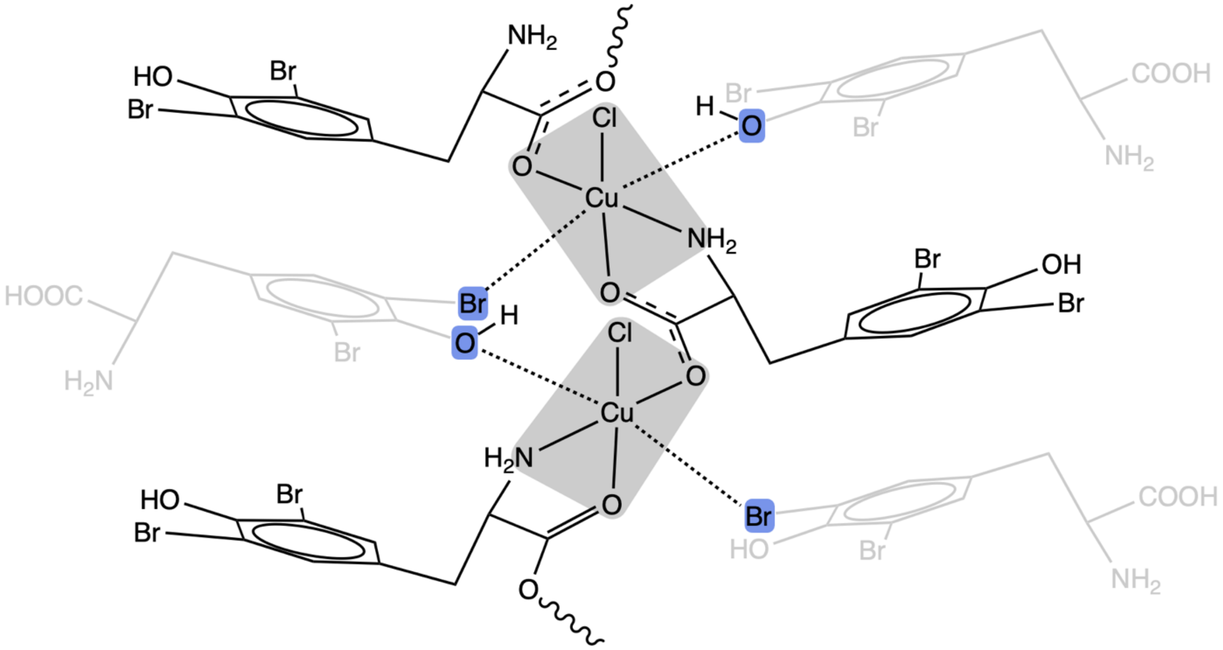

Scheme S1. Scheme of the fragment of {[CuCl(μ–*O,O'*–L–Br_2Tyr)]}$_n$ polymer structure cut out from crystal lattice and used in theoretical calculations (AIM, NCI, NBO).
